# The role of salivary vesicles as a potential inflammatory biomarker to detect traumatic brain injury in mixed martial artists

**DOI:** 10.1038/s41598-021-87180-4

**Published:** 2021-04-14

**Authors:** Rani Matuk, Mandy Pereira, Janette Baird, Mark Dooner, Yan Cheng, Sicheng Wen, Shyam Rao, Peter Quesenberry, Neha P. Raukar

**Affiliations:** 1 Warren Alpert Medical School, Brown University, Providence, RI USA; 2 Division of Hematology/Oncology, Department of Medicine, Rhode Island Hospital, Providence, RI 02903 USA; 3 Department of Emergency Medicine, Rhode Island Hospital, Providence, RI USA; 4 Division of Neurology/Neurocritical Care, Department of Medicine, Rhode Island Hospital, Providence, RI USA; 5 Department of Emergency Medicine, Mayo Clinic, Rochester, MN USA

**Keywords:** Biological techniques, Biotechnology, Neuroscience, Biomarkers, Molecular medicine, Neurology

## Abstract

Traumatic brain injury (TBI) is of significant concern in the realm of high impact contact sports, including mixed martial arts (MMA). Extracellular vesicles (EVs) travel between the brain and oral cavity and may be isolated from salivary samples as a noninvasive biomarker of TBI. Salivary EVs may highlight acute neurocognitive or neuropathological changes, which may be particularly useful as a biomarker in high impact sports. Pre and post-fight samples of saliva were isolated from 8 MMA fighters and 7 from controls. Real-time PCR of salivary EVs was done using the TaqMan Human Inflammatory array. Gene expression profiles were compared pre-fight to post-fight as well as pre-fight to controls. Largest signals were noted for fighters sustaining a loss by technical knockout (higher impact mechanism of injury) or a full match culminating in referee decision (longer length of fight), while smaller signals were noted for fighters winning by joint or choke submission (lower impact mechanism as well as less time). A correlation was observed between absolute gene information signals and fight related markers of head injury severity. Gene expression was also significantly different in MMA fighters pre-fight compared to controls. Our findings suggest that salivary EVs as a potential biomarker in the acute period following head injury to identify injury severity and can help elucidate pathophysiological processes involved in TBI.

## Introduction

Several studies have looked retrospectively at the types of injuries sustained through mixed martial arts (MMA)^1–8^, though these reviews are based on different time periods or “eras” of MMA. Regulation has improved since the 1990s, when nascent forms of cage fighting with minimal protective equipment first arose in the US to more recent professional MMA events with unified regulations^5^. Sustained injuries are commonly orthopedic in nature, such as fractures, and include simple and complex lacerations^2,3,5,6,9^. A major concern is head trauma and cognitive impairment, including traumatic brain injury (TBI) or even death. This is not unique to MMA but has been the source of contention for many contact sports, particularly with the prevalence of chronic traumatic encephalopathy (CTE)^10^ resulting from repetitive hits to the head over time^4,11–14^. Boxing has long been referenced for its connection to dementia^15–17^ (dementia pugulistica) and physiological change as a result of repetitive direct hits to the head. MMA injury patterns are closest to those of boxing^1,6,18^.

Mild TBI (mTBI) typically incorporates concussion, which most frequently does not involve loss of consciousness. The implications of concussion are numerous, particularly in the adolescent population, with measured deficiencies in cognition, balance, mood, and memory seen within 24 h of injury^19^. Popular “sideline” tests for concussion include the SCAT5 (Sports Concussion Assessment Tool), King-Devick, and SAC (Standardized Assessment of Concussion), which often rely on subjective indices^20^. Other forms of office based testing include neuropsychiatric assessment, though this may have a skill bias. In addition, patients with existing learning disabilities may complicate the presentation and interpretation of concussive symptoms and testing^21–23^. Currently no objective, biological test exists to quickly determine the presence or severity of a concussion^24–27^.

Extracellular vesicles (EVs) are cell derived vesicles that function as mediators of cell-to-cell communication and can be readily isolated in biofluids^24,28–34^. The contents include functional mRNA transcripts subject to translation and microRNA which may regulate post transcriptional gene expression, alter the proteome and influence processes in distant recipient cells^35^. The complex molecular composition of EVs respond to stimuli and stability provided by a secure lipid membrane to fill the gap of known limitations of extracellular protein and nucleic acid biomarkers^36,37^.

There is a connection between salivary RNA and severity and duration of concussive symptoms in pediatrics^35^ and in pediatric subjects with a confirmed TBI and salivary EVs have shown promise as a possible marker for TBI^38^.

The goal of this study was to evaluate acute changes in salivary EVs gene expression in subjects before and after a head injury. Salivary EVs from mixed martial artists before and after fights (stressor event) were analyzed to identify changes in expression of inflammation related genes.

## Materials and methods

### Subject selection and fight characteristics

Participants in the study consisted of male and female mixed martial artists aged 18–30 recruited through an officially sanctioned amateur and professional mixed martial arts event. The review of research for human subjects was granted by the Rhode Island Hospital Lifespan Institutional Review Board (IRB #4093–16). All participants and healthy control participants gave written informed consent. All clinical investigations have been conducted according to the principles expressed in the Declaration of Helsinki and have been carried out according to the international Good Laboratory Practice (GLP) and Good Clinical Practice (GCP) standard. No personal medical information was taken or used in publication. Participant samples were deidentified.

Prior to the start of the event, all fighters underwent background checks and a medical examination. Professional fighters additionally provided evidence of a normal brain CT, brain MRI, or neurological examination within 5 years.

Saliva samples were obtained from 20 fighters (20 pre-fight and 17 post-fight). Of these,16 fighter samples (8 pre-fight and 8 post-fight) were selected for analysis based on available gene marker cards and quality of samples. In addition, 7 control samples from non-fighters were analyzed.

Fight results were recorded and included outcome or mechanism of stoppage, as well as length of time of fight, lasting up to 3 rounds per match. Data was verified by officials following the conclusion of the event. Any loss of consciousness, if applicable, was also recorded. The mechanism of stoppage was recorded as Judges’ Decision, No-Contest, Submission (Choke or Joint Lock), Knockout (KO), or Technical Knockout (TKO) due to referee stoppage.

Control samples of saliva collected from non-head trauma participants are summarized in Table 1. Table 2 summarizes fight information including gender, loss of consciousness, fight outcome, round of fight completion, method of win/loss, and mechanism of injury if a fight ended prior to decision.

**Table 1:** Summary of control participant group.

Control	Gender	Age
1	F	22
2	F	22
3	F	25
4	F	33
5	F	23
6	M	22
7	M	N/A

**Table 2:** Summary of Fighter Data: gender (male/female), outcome (win(W)/lose(L)/draw(D)/no contest(NC)), round of fight completion (1/2/3), method of outcome (Knockout(KO)/Technical Knockout(TKO)/Submission(S)/Judges’ Decision (D), Mechanism (P = punches, J = joint decision, C = choke, NA = not applicable), loss of consciousness (LOC = Yes/No).

Fighter	Gender	Age	Outcome	Round	Method	Mechanism	LOC
1	M	18–30	W	1	S	J	N
2	M	18–30	L	2	TKO	P	N
3	M	18–30	W	1	S	C	N
4	F	18–30	W	3	D	NA	N
5	M	18–30	W	3	D	NA	N
6	F	18–30	L	2	TKO	P	N
7	F	18–30	W	2	TKO	P	N
8	M	18–30	L	3	TKO	P	N

### Saliva sample collection

Each subject provided approximately 5 mL of saliva into a 50 mL tube within 4 h before the fight (pre-fight sample), followed by an approximate 5 mL sample of saliva into a separate 50 mL tube within 1 h after the fight (post-fight sample).

Saliva samples were stored on ice for the duration of the event and were transferred to storage in dry ice or − 80°Celsius (C) within 1 h of the conclusion of the event. The collected pre- and post-fight samples were then immediately transferred to − 80 °C storage.

### EV isolation

The human saliva EVs were isolated by differential centrifugation^39^ using lab protocol^40,41^. Briefly, each saliva sample was mixed with an equal volume of phosphate buffered saline (PBS) and centrifuged at 5000 g for 20 min at 4 °C. The supernatant was ultracentrifuged at 12,000 g for 30 min also at 4 °C. The supernatant from previous centrifugation was ultracentrifuged at 120,000 g for 70 min at 4 °C. EV pellets were re-suspended in 1% dimethyl sulfoxide in PBS for long term storage at − 80 °C.

### EV quantity and size characterization by nanosight

EVs quantification was done by Nanosight NS500 (Malvern). The analysis settings calibrated using control beads, then kept constant for all samples. Each video gives an estimated concentration, mean size, with standard deviation. Samples were diluted 1:20.

### Transmission electron microscopy

EM done using a modified uranyl acetate replacement staining protocol^42^. Briefly, EVs were absorbed onto formvar/carbon copper grids (Electron Microscopy Sciences) then stained with Uranyl acetate replacement solution (Electron Microscopy Sciences) then stained with 3% lead citrate Reynolds Stain (Electron Microscopy Sciences). Images taken on Philips 410 transmission electron microscope with Advantage HR CCD camera.

### Western blot analysis

Protein concentration measured by bicinchoninic acid (BCA) protein array (Pierce). EVs were digested with RIPA buffer and 20 µg of EVs lysates were separated by 4–20% MP TGX Stain-Free Gel (Bio-Rad) electrophoresis and proteins were transferred to membranes using the Trans-Blot Turbo (Bio Rad). Primary antibodies including CD63, CD81, CD9, and heat shock protein 70(hsp70) from System Biosciences EXOABPKIT-1, apoA-I (Santa Cruz Biotechnology), GAPDH (Invitrogen), PDCD6IP (ThemoFisher Scientific) were used for Western Blot.

### RNA isolation and quantification

To isolate the RNA, the EV pellets were lysed with Trizol (Invitrogen), RNA isolation as recommended by the manufacturer. Chloroform samples were added to the sample and centrifuged to collect RNA in the aqueous phase, which was suspended in RNase free water. The NanoDrop 1000 (ThermoFisher) was used for RNA quantification.

### Real time PCR analysis

Reverse transcription was completed with a high capacity cDNA transcription kit (Life Technologies) according to the manufacturer. RNA concentration for all samples was between10 and 50 ng. cDNA amplification process was one cycle of 10 min at 25 °C, one cycle of 120 min at 37 °C, and one cycle of 5 min at 85 °C using a 9800 Fast Thermal Cycler. The pre-amplification reactions were done using TaqMan Preamp Master mix (Life Technologies) according to manufacture guidelines in a final volume of 50 µl. Samples were tested prior to array loading. Each sample was tested with endogenous genes. Samples yielding consistent CT values to each endogenous gene were used.

The TaqMan Human Inflammation (Life Technologies) card was used for gene expression analysis. The array card contained 92 genes associated with inflammatory response with 4 endogenous controls as in Supplemental Table S1. Array cards were loaded with cDNA and TaqMan Universal PCR Master Mix (Life Technologies) and analyzed on a Viia7 Real-Time PCR System (Life Technologies). Cycle threshold (CT) readings were used to interpret fold change in gene expression. Average pre and post CT values were then calculated and further filtered at a cut-off of CT 35 at the aggregate level—the industry standard. Average level filtering was used, rather than individual level filtering. Delta delta CT (ddCT) was used to calculate the relative fold gene expression of samples^43,44^. Raw data CT values for MMA fighters on Supplemental Table S2 and raw data CT values for non-fighter controls on Supplemental Table S3.

### Statistical analysis methods

#### Two-tailed confidence interval

Data demonstrated minimal skewness and kurtosis with values less than + / − 1. Furthermore, normalized data plotted demonstrated a generally uniform distribution when compared to a Gaussian curve. Given these estimates, we used a 95 percent confidence interval adjusted for sample standard error to create a filter for extreme values at the ddCT level. Null hypothesis was defined as a ddCT equal to 0 to imply no change between pre and post samples. Data that fell outside of these ranges was considered extreme and flagged for further consideration. With the extreme values, we used the 2 exp(-ddCT)^43^ method to determine relative gene expression with up-regulation noted at a value of greater than 2 and down-regulation noted at a value less than 0.5. A value of 1.0 would represent similar upward and downward expression.

#### One-way ANOVA

EV characteristics, mean size and concentration, of the three participant groups were compared using ANOVA. *P*-value under 0.05 was considered significant.

#### Wilcoxin signed rank test

Paired samples of pre- and post-fight data were compared. A filter using the Wilcoxin signed-rank test for non-parametric analysis was used. For this filter, we used an alpha of 0.05 and 0.10 for robustness. Critical values were 3 and 5, respectively, given the paired sample size of 8 pre and 8 post samples. Genes with Wilcoxin values less than or equal to these cut-offs were included as outlier genes.

### Gene pathways

The KEGG database was used for pathway analysis^45^. The pathways were then categorized into type and class to review common patterns with the final list of genes based on available functional data^46^.

## Results

### EV characteristics

**Figure 1:**
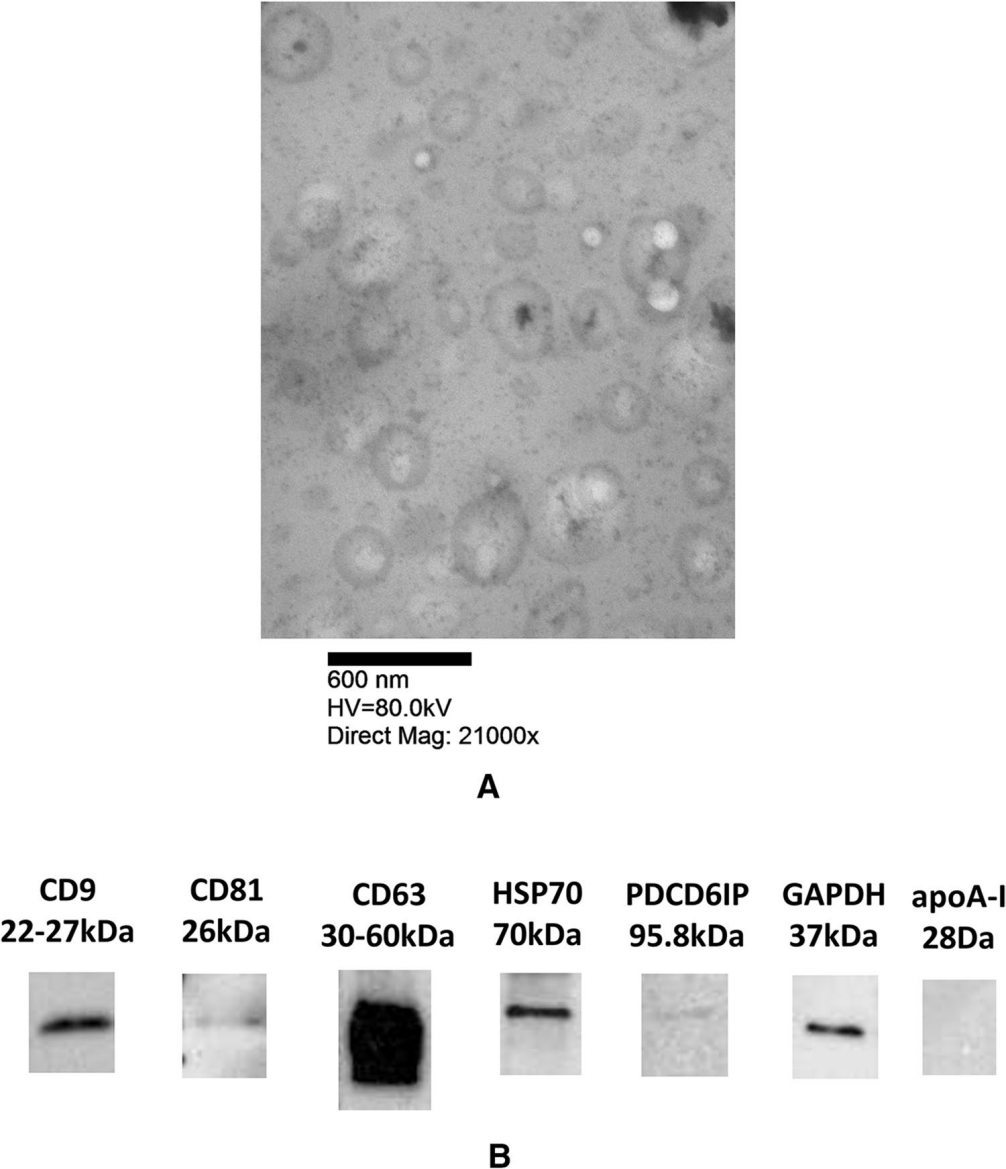
TEM image of salivary EV, 21000 K (**A**). Representative image of salivary EV western blot. Western blot analysis of samples shows the presence of EV markers: CD9, CD63, CD81, HSP70, PDCD6IP, and negative for apoA-I (**B**).

The Society for Extracellular vesicles has outlined general characteristics for EV determination^47^. In this study we utilize, transmission electron microscopy (TEM), Nanosight, and Western blot analysis to characterize the salivary EVs. TEM was done on the saliva EV samples shows range of sizes from 50 to 1000 nm (Fig. 1A). Western blot analysis of samples shows the presence of EV markers: CD9, CD63, CD81, HSP70, PDCD6IP, but not apoA-I (Fig. 1B; Full western blot Supplemental Fig. S1). Nanosight analysis, mean size and concentration, of fighter EV samples and control EV samples shown in Supplemental Fig. S2. EV mean size was 333 nm for the control participants, 282 nm for MMA fighters pre-fight, and 258 nm for MMA fighters post-fight. Anova analysis of EV size showed no statistical difference between the three groups, *p*-value of 0.16. EV concentration was 3.86 × 10^10^ for the control participants, 2.01 × 10^10^ for MMA fighters pre-fight, and 4.55 × 10^10^ for MMA fighters post-fight. Anova analysis of concentration showed no statistical difference between the three groups, *p*-value of 0.45.

### Gene filtering

Seven significant key genes, ALOX5, HRH1, PDE4B, ADRB2, ANXA1, ITGB2, and MAPK8, passed three levels of testing (Table 3). Group 1 had three genes that passed the Wilcoxin filter test at a 0.05 alpha level. Group 2 included an additional four genes (7 total) out of 92 starting genes that passed the Wilcoxin filter test a 0.10 alpha level. Group 3 was used as a confirmation check and included 31 genes (including Groups 1 and 2) that passed the Confidence Interval filter test at a 0.05 alpha level. The seven overlapping genes of Groups 1 and 2 were then used as the final analysis list for further pattern recognition.

**Table 3:** List of filtered genes by statistical method.

Group 1	Group 2	Group 3	Gene full name
Wilcoxin .05	Wilcoxin .10	CI 0.05	
		ADRB1	Adrenoreceptor Beta 1
	**ADRB2**	**ADRB2**	Adrenoreceptor Beta 2
**ALOX5**	**ALOX5**	**ALOX5**	Arachidonate-5-Lipoxygenase
		ALOX12	Arachidonate-12-Lipoxygenase
	**ANXA1**	**ANXA1**	Annexin A1
		ANXA3	Annexin A3
		CASP1	Caspase 1
		CYSLTR1	Cysteinyl Leukotriene Receptor 1
		HPGD	15-Hydroxyprostaglandin Dehydrogenase
**HRH1**	**HRH1**	**HRH1**	Histamine Receptor H1
		HRH2	Histamine Receptor H2
		ICAM1	Intercellular Adhesion Molecule 1
		IL1R2	Interleukin 1 Receptor Type 2
		ITGB1	Integrin Subunit Beta 1
	**ITGB2**	**ITGB2**	Integrin Subunit Beta 2
		KLK1	Kallikrein 1
		LTA4H	Leukotriene A4 Hydrolase
		LTB4R2	Leukotriene B4 Receptor 2
		LTB4R	Leukotriene B4 Receptor
		MAPK14	Mitogen-Activated Protein Kinase 14
		MAPK3	Mitogen-Activated Protein Kinase 3
	**MAPK8**	**MAPK8**	Mitogen-Activated Protein Kinase 8
		NFKB1	Nuclear Factor Kappa B Subunit 1
**PDE4B**	**PDE4B**	**PDE4B**	Phosphodiesterase 4B
		PDE4D	Phosphodiesterase 4D
		PTAFR	Platelet Activating Factor Receptor
		PTGDR	Prostaglandin D2 Receptor
		TBXAS1	Thromboxane A Synthase 1
		TNF	Tumor Necrosis Factor
		TNFRSF1A	TNF Receptor Superfamily Member 1A
		TNFRSF1B	TNF Receptor Superfamily Member 1B

Group 1 genes with *p* value < 0.05, Group 2 genes with *p* value < 0.10, Group 3 genes with a confidence interval of 0.05.

### Comparison between pre and post fight samples

**Figure 2:**
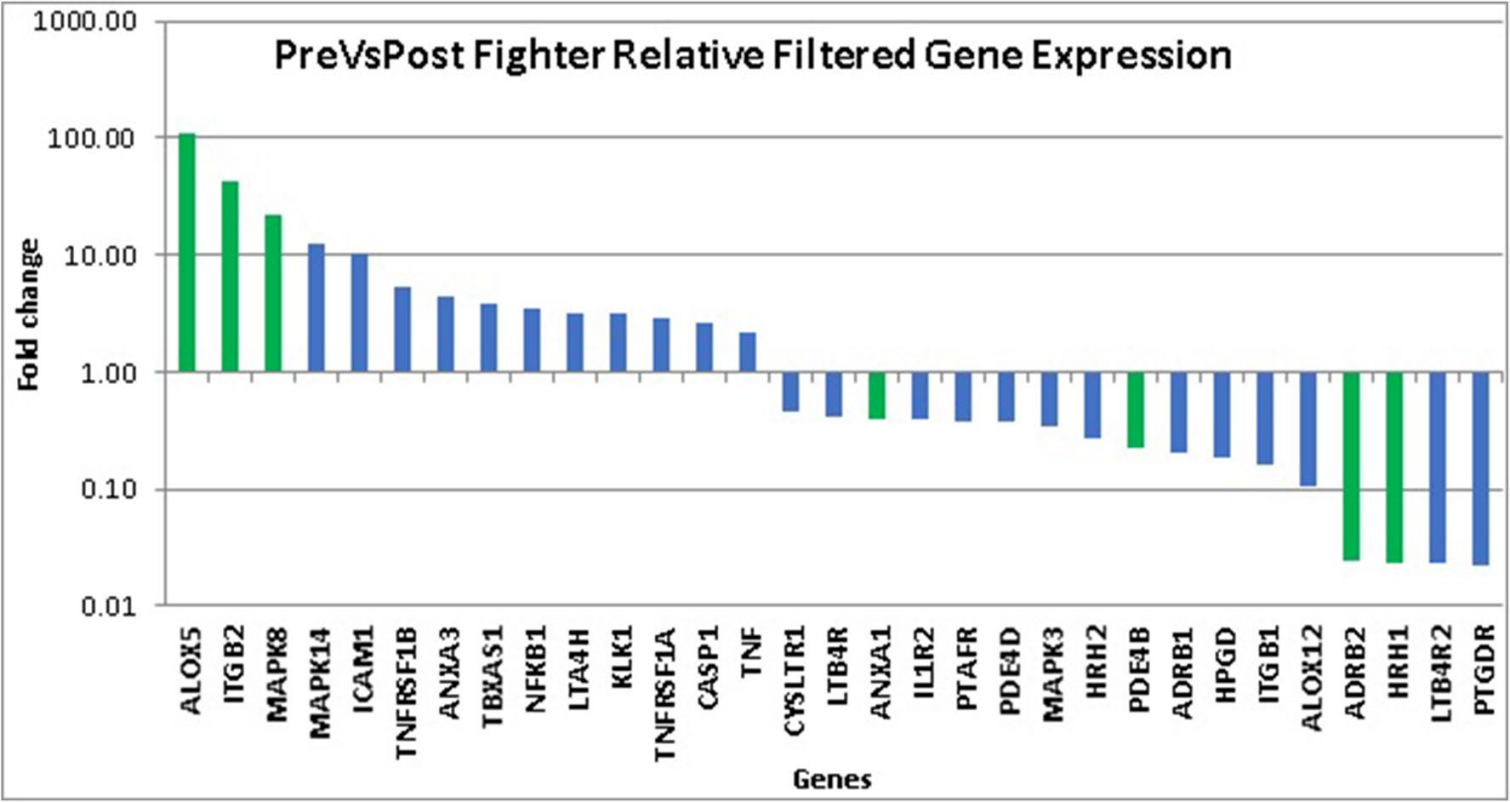
Pre fighter versus post fighter relative filtered gene expression—fold change. Seven key genes are highlighted in green.

The relative expression of filtered genes was charted for significant changes from pre to post fight for each sample (Fig. 2). ALOX5, ITGB2, and MAPK8 demonstrated the highest upregulation, with ALOX5 at 111-fold change. ADRB2 and HRH1 were downregulated at an average fold change of 0.02.

### Comparison between control and fight samples

**Figure 3:**
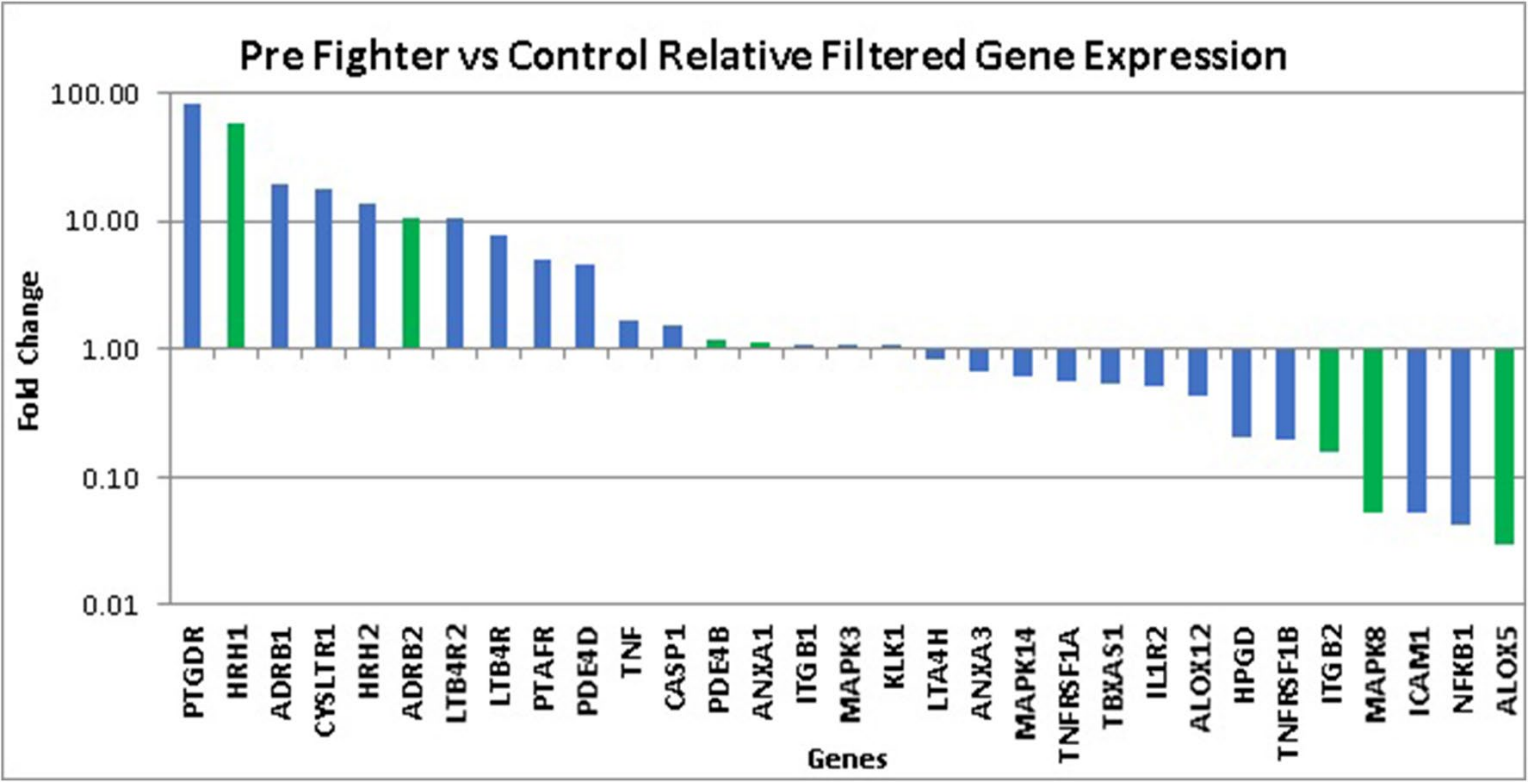
Pre fighter versus control relative filtered gene expression—fold change. Seven key genes are highlighted in green.

The relative expression of the final list of filtered genes from pre-fight samples was compared to healthy non-head trauma controls to study baseline changes in gene expression prior to the start of the fight. The seven key genes are shaded green from the group of 31 genes as shown in Fig. 3. HRH1 demonstrated a 58-fold upregulation, while ALOX5 was downregulated 0.03-fold.

### General fighter gene attribution

We examined the effect of fighting on gene expression by using a standardized signal of changes in pre-fight versus post-fight expression normalized by standard deviation as an absolute value [Median ddCT/St Dev ddCT]. This was completed at the 31 gene level after a Confidence Interval filter at the 0.05 alpha level and at the seven key gene level after a Wilcoxon filter test at 0.19. Table 4 displays the components and ranking of these signals at the filtered 31 and key seven gene levels.

**Table 4:** Standardized gene signals of filtered genes where signal is absolute value [Median ddCT/St Dev ddCT].

Fighter	Median	SD	Signal31	Rank31	Median	SD	Signal7	Rank7
	DDCT_31	DDCT_31			DDCT_7	DDCT_7		
1_MMA	− 0.76	4.80	0.16	7	0.29	6.04	0.05	8
2_MMA	1.81	4.73	0.38	1	1.84	0.25	7.47	1
3_MMA	3.93	5.69	0.69	6	0.52	7.82	0.07	6
4_MMA	1.11	4.16	0.27	4	1.60	10.17	0.16	4
5_MMA	0.64	5.07	0.13	3	1.39	5.25	0.26	2
6_MMA	− 0.39	6.59	0.06	5	− 0.88	6.67	0.13	5
7_MMA	0.46	6.06	0.08	8	− 0.65	12.23	0.05	7
8_MMA	1.82	4.14	0.44	2	1.22	5.93	0.21	3

Signal of 31 genes with a confidence interval of 0.05. Signal of 7 genes with *p* value < 0.10.

At the 31 gene level, the largest signals were for fighter 2 (male, loss by TKO by punches), fighter 8 (male, loss by TKO by punches), and fighter 5 (male, won by decision). The lowest signals were for fighter 7 (male, won by TKO by punches), fighter 1 (male, won by joint submission), and fighter 3 (male, won by choke).

At the seven gene level, the largest signals were for fighter 2 (male, loss by TKO by punches), fighter 5 (male, won by decision), and fighter 8 (male, loss by TKO by punches). The smallest signals were for fighter 1 (male, won by joint submission), fighter 7 (male, won by TKO by punches), and fighter 3 (male, won by choke).

**Figure 4:**
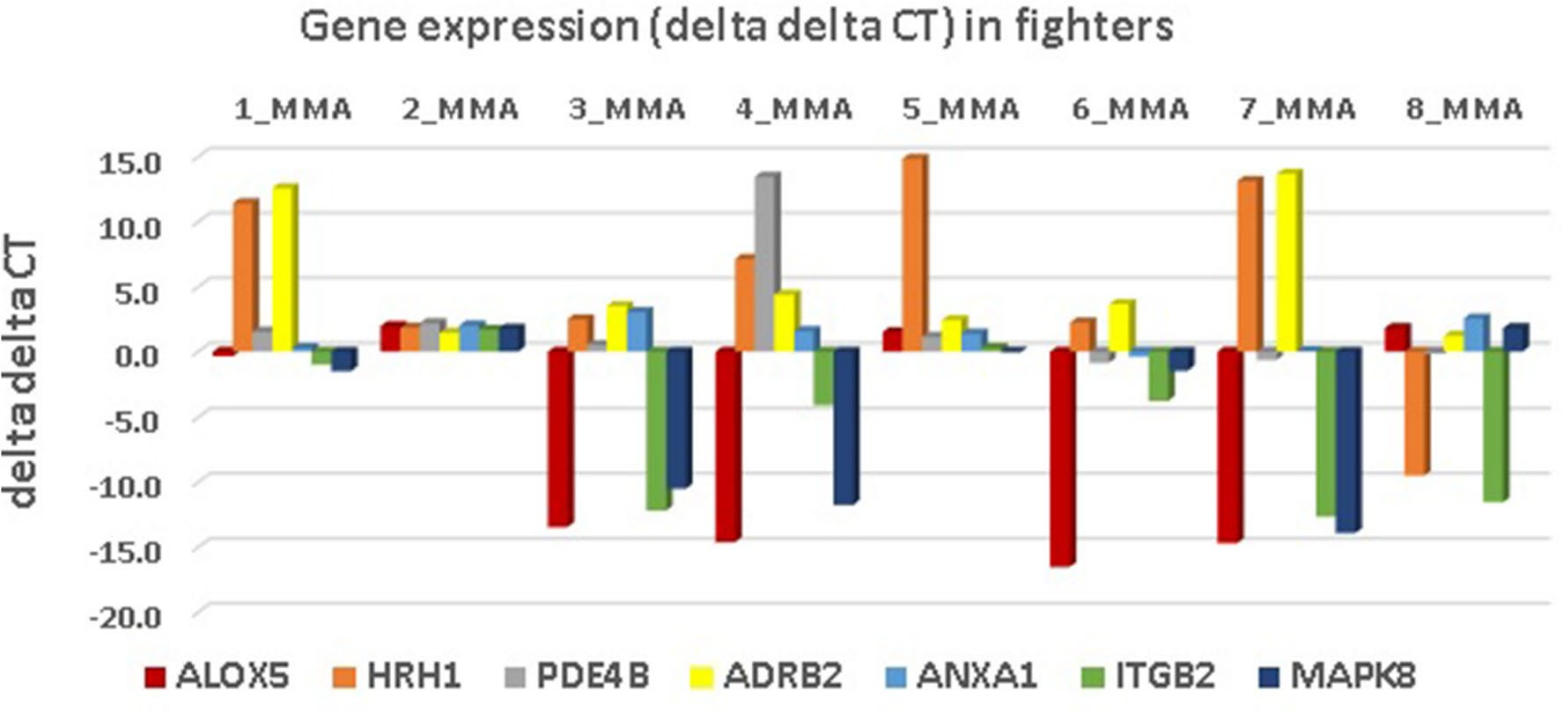
Fighter Level Delta Delta CT (cycle threshold) for seven key genes.

The ddCT of the seven key genes for each fighter is shown in Fig. 4. The seven significant (*p* = 0.10) genes with the largest upregulation (ALOX5, ITGB2, and MAPK8) were driven by a predominance of negative ddCT values across all fighters, while the genes with the largest downregulation (ADRB2, HRH1) were driven by a predominance of positive ddCT values across all fighters.

### Gene pathways and classes

The 31 filtered genes were analyzed to create a database of biosynthesis pathways and maps^45^ which was then categorized based on function in the inflammatory pathway. Of the 130 potential unique inflammatory pathways, the 31 genes most commonly affected were involved in 29 of these pathways, often present in multiple pathways. Signal Molecules and Interaction and Signal Transduction were the two most commonly affected classes of gene expression, and further analysis showed eight genes (26%) appeared in the Neuroactive Ligand-Receptor Interaction pathway and seven genes (23%) appeared in the Calcium Signaling pathway with at least one of the seven key genes involved in Arachidonic Acid Metabolism, Serotonergic Synapse, Cell Adhesion Molecules, cAMP Signaling, Rap1 signaling pathway, cGMP-PKG Signaling, Phagosome, and Hippo signaling pathway^45^. Supplemental Table S4 summarize the pathways, classes, and genes associated with TBI.

## Discussion

When selecting fighter samples for gene analysis, we focused on mechanism of injury. We hypothesized that athletes who had more cumulative hits in longer fights (decision or later round matches) and more severe mechanisms and methods of injury (knockout or technical knockout by punches or kicks) would have higher genetic expression than those who had shorter bouts or less hits. We then analyzed signal changes in pre-fight versus post-fight expression normalized by standard deviation as an absolute value [Median ddCT/St Dev ddCT]^43^. Using this standard technique, we noted the highest ratios in fighters sustaining longer fights and those who sustained a technical knockout. Conversely, we noted the lowest ratios for fighters who fought the least amount of time and those who won matches in earlier rounds. This result was particularly evident at the key seven gene level and suggests a pattern of higher genetic signal associated with mechanism of injury.

The filtered genes that were identified frequently belonged to classes of signal transduction with common pathways of neuroactive ligand-receptor interactions, calcium signaling pathways, and arachidonic acid metabolism^45^. These were highlighted by relative gene expression for the fighters when comparing pre-fight versus post-fight and pre-fight versus control, suggesting an upregulation even at baseline in these athletes that is further expressed in the acute stage of injury. The mediator cyclooxygenase-2 and resulting metabolites and free radicals/reactive oxygen species that are present during the conversion of arachidonic acid to both classic and novel prostaglandins have all shown connections to TBI and neurodegeneration via increased neuroinflammation and lipid peroxidation^48–52^. Also, various metabolites of arachidonic acid play a role in the breakdown of the blood brain barrier suggesting a mechanism for further or secondary injury following TBI^53^. Calcium signaling plays a critical role in neurotransmission and lipid peroxidation mediated buffering errors which leads to a disruption in signaling processes, compounding neuronal damage^54^.

The 7 key genes affected include ALOX5, ITGB2, MAPK8, ANXA1, PDE4B, ADRB2, and HRH1. ALOX5 is specifically involved with catalyzing arachidonic acid to leukotrienes and various eicosanoids, which are significant mediators of multiple inflammatory systems^46^. ITGB2 is an integrin involved with cell adhesion and surface signaling^46^. MAPK8 is a mitogen-activated protein kinase activated by TNF alpha for mediation of cell death and apoptosis as well as T cell proliferation^46^. ADRB2 is a beta adrenoreceptor frequently involved with mediation of catecholamines and is connected to L-type calcium channels (long-lasting voltage-dependent)^46^. HRH1 is a histamine receptor and messenger molecule from mast cells that is significantly involved with neurotransmission in the CNS^46^. ANXA1 is a phospholipase inhibitor and inflammatory regulator that blocks eicosanoid production and inhibits various leukocyte events such as adhesion, chemotaxis, and phagocytosis^46^. PDE4B is involved in regulation of cyclic nucleotide concentration and signal transduction. Furthermore, it negatively affects cAMP activity, which is a powerful physiological regulator for intracellular signal transduction^46^. PDE4B inhibitors have shown promise in TBI by increasing cAMP and its associated proteins connected to memory and learning^55^. Reduced expression of PDE4B post-fight may be associated with protective mechanisms against cognitive decline in TBI^55^.

The genes that were found to upregulate the most during a fight were ALOX5, ITGB2, and MAPK8, and notably, all three baseline values were downregulated when comparing pre-fight samples to control data. ALOX5 plays a role in lipid signaling and is connected to physiological stress induction^46^ and the significant upregulation of ALOX5 post-fight serves as an apparent signal of physical stress. Pre-fight samples however had low levels of ALOX5 when compared to non-head trauma controls. We can postulate that fighters developed adaptive responses to chronic physiologic stress resulting in baseline ALOX5 downregulation with significant fight related impact overwhelming these mechanisms leading to acute post-fight ALOX5 upregulation. Further study correlating clinical indicators of physiologic/psychological stress with ALOX5 expression are needed to elucidate this relationship.

The genes that were found to downregulate the most during a fight were ADRB2, HRH1, ANXA1 and PDE4B, with the first two upregulated at baseline for pre-fight samples when compared to control data and the latter two with pre-fight gene expression similar to non-head trauma controls.

Our study demonstrates the feasibility of salivary EVs as a potential for acute biomarkers in head injury.

## Limitations and further study

Our study was limited by its small sample size and that the preponderance of male fighters. Future studies should explore gene expression based on age, sex, skill level, mechanism of trauma, and duration of the contest. While we did find genes that were up- and downregulated when compared to pre-fight levels and when compared to controls, the clinical relevance of these genes will need to be further investigated. Finally, studies with a larger sample size and correlating salivary EV gene expression with long term cognitive outcomes are needed.

## Conclusions

This study demonstrated feasibility of salivary EVs analysis in mixed martial artists before and after a fight. We found several patterns of key gene expression and pathways affected acutely and chronically after a head injury. Using a standardized information ratio of genetic expression, we were able to demonstrate correlation between suspected damage and signal strength across our samples. This suggests promise as an objective test of neuronal damage. Prior studies compared EVs of confirmed injured patients to a control group, rather than using each subject as his or her own control. These gene expression and pathway patterns and commonalities further demonstrate the use of saliva as a viable mechanism for elucidating TBI or inflammation. This may help with early diagnosis of TBI and may guide more quantitatively driven return to play decisions^56,57^. Furthermore, it may demonstrate some protective genetic patterns inherent to an athlete. The idea of a noninvasive biomarker of TBI presence or severity is an exciting new area of research.

## Supplementary Information

Supplementary Information 1.
