# Supplementary material for: The role of salivary vesicles as a potential inflammatory biomarker to detect traumatic brain injury in mixed martial artists
Source: Sci Rep. 2021 Apr 14;11:8186. doi: 10.1038/s41598-021-87180-4 (PMC8047010; doi:10.1038/s41598-021-87180-4)
Supplement: Supplementary file 1 — Supplementary Information 1. [file 41598_2021_87180_MOESM1_ESM.docx]

The role of salivary vesicles as a potential inflammatory biomarker to detect traumatic brain injury in mixed martial artists

Rani Matuk_­_­^1^, Mandy Pereira^2^, Janette Baird^3^, Mark Dooner^2^, Yan Cheng^2^, Sicheng Wen^2^, Shyam Rao^4^, Peter Quesenberry^1,2^, Neha P. Raukar^3,5^

^1^Warren Alpert Medical School of Brown University, Rhode Island Hospital, Providence, RI

^2^Department of Medicine Division of Hematology/Oncology, Rhode Island Hospital, Providence, RI
^3^Department of Emergency Medicine, Rhode Island Hospital, Providence, RI

^4^Department of Medicine Division of Neurology, Rhode Island Hospital Providence RI

^5^Department of Emergency Medicine, Mayo Clinic, Rochester, MN

**Supplemental Table S1.** Taqman Human Inflammation array. List of genes with catalog number from Life Technologies.

**
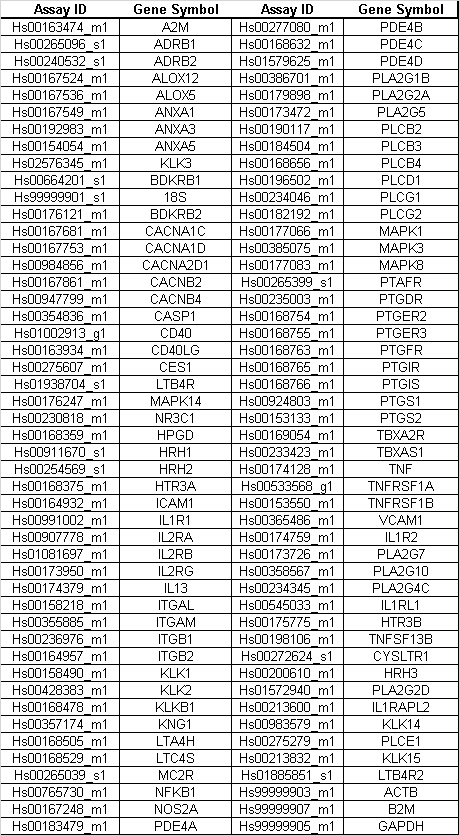
**

**Supplemental Table S2**. Cycle threshold values from real time PCR for 8 players (16 samples: 8 pre fight match and 8 post fight match) for 96 genes from the Taqman Human Inflammation array. U= undetermined

| **Player** | **MMA1** | | **MMA2** | | **MMA3** | | **MMA4** | | **MMA5** | | **MMA6** | | **MMA7** | | **MMA8** | |
| --- | --- | --- | --- | --- | --- | --- | --- | --- | --- | --- | --- | --- | --- | --- | --- | --- |
| **time** | **pre** | **post** | **pre** | **post** | **pre** | **post** | **pre** | **post** | **pre** | **post** | **pre** | **post** | **pre** | **post** | **pre** | **post** |
| **Sample** | **M01** | **M15** | **M35** | **M02** | **M05** | **M06** | **M37** | **M31** | **M22** | **M13** | **M09** | **M16** | **M08** | **M07** | **M18** | **M33** |
| **18S** | 24.182 | 24.165 | 21.516 | 18.094 | 27.576 | 24.802 | 25.423 | 25.687 | 18.052 | 21.390 | 30.386 | 23.344 | 24.976 | 24.673 | 29.078 | 32.149 |
| **A2M** | U | U | U | U | U | U | U | U | 28.866 | 26.327 | U | U | U | U | U | U |
| **ACTB** | 22.406 | 23.802 | 19.690 | 17.303 | 26.038 | 21.720 | 24.421 | 23.294 | 16.529 | 20.283 | 22.633 | 20.394 | 22.648 | 23.032 | 25.345 | 24.843 |
| **ADRB1** | 28.283 | U | 25.025 | 23.178 | 24.015 | 23.726 | 25.462 | 27.977 | 24.190 | 28.482 | 25.063 | 27.278 | U | 32.238 | 28.998 | 28.599 |
| **ADRB2** | 26.666 | U | 23.984 | 21.848 | 21.966 | 21.545 | 22.441 | 25.711 | 22.717 | 27.460 | 23.106 | 26.762 | 26.780 | U | 28.055 | 27.454 |
| **ALOX12** | 28.801 | 27.780 | 24.518 | 22.505 | 27.368 | 27.985 | 27.499 | 27.330 | 21.984 | 28.789 | 25.957 | U | 26.649 | 26.256 | U | U |
| **ALOX5** | 26.032 | 26.448 | 23.280 | 21.673 | U | 22.610 | U | 24.276 | 19.511 | 23.332 | U | 23.475 | U | 24.858 | U | U |
| **ANXA1** | 22.629 | 23.681 | 18.358 | 16.812 | 23.038 | 22.144 | 22.397 | 22.887 | 16.525 | 20.233 | 21.723 | 21.331 | 22.494 | 22.086 | 25.399 | 26.135 |
| **ANXA3** | 26.242 | 29.014 | 25.081 | 22.979 | 29.807 | 26.259 | U | 27.752 | 21.953 | 24.920 | 27.200 | 24.487 | 27.760 | 27.241 | U | 30.631 |
| **ANXA5** | 24.915 | 24.435 | 23.217 | 21.379 | U | 27.534 | 26.152 | 26.672 | 20.919 | 22.758 | 27.393 | 24.591 | 26.467 | 26.422 | 28.164 | 28.479 |
| **B2M** | 22.120 | 22.471 | 18.358 | 16.191 | 23.021 | 18.750 | 22.061 | 20.986 | 15.857 | 19.401 | 21.659 | 20.224 | 23.151 | 21.644 | 24.778 | 23.291 |
| **BDKRB1** | U | 35.314 | 23.610 | 21.798 | 22.914 | 22.335 | 23.811 | 26.493 | 22.757 | 27.174 | 24.640 | 29.069 | 28.870 | 28.999 | U | 28.446 |
| **BDKRB2** | U | U | U | 23.398 | U | U | U | U | U | U | U | 24.916 | 33.044 | U | U | U |
| **CACNA1C** | U | U | U | 24.909 | U | U | U | U | 26.723 | U | U | 33.721 | U | U | U | U |
| **CACNA1D** | U | U | U | 28.863 | U | U | U | U | 29.318 | U | U | 38.444 | U | U | U | U |
| **CACNA2D1** | U | U | U | U | U | U | U | U | 25.423 | 36.922 | U | 36.526 | U | U | U | U |
| **CACNB2** | U | U | U | U | U | U | U | U | 28.296 | U | U | U | U | U | U | U |
| **CACNB4** | 29.449 | 29.999 | 26.776 | 26.546 | U | 29.848 | U | 29.809 | 25.548 | 27.437 | U | 29.293 | U | U | U | U |
| **CASP1** | 28.959 | 28.810 | 27.154 | 23.758 | 29.067 | 25.895 | 28.776 | 27.180 | 22.309 | 25.295 | 26.692 | 26.291 | 28.776 | 26.963 | U | 28.763 |
| **CD40** | U | U | U | 26.220 | U | U | U | U | 25.483 | 28.311 | U | 26.883 | U | U | U | U |
| **CD40LG** | U | U | U | 36.124 | U | U | U | U | 30.032 | U | U | 36.091 | 35.614 | U | U | U |
| **CES1** | U | U | 34.500 | 28.103 | U | U | 29.730 | U | 26.888 | U | U | U | U | U | U | U |
| **CYSLTR1** | 28.491 | 27.324 | 25.441 | 24.976 | 24.852 | 23.604 | 24.515 | 27.603 | 24.060 | 25.609 | 28.769 | 29.051 | 26.985 | 25.975 | U | U |
| **GAPDH** | 23.653 | 24.414 | 21.579 | 18.009 | 27.052 | 23.119 | 25.924 | 24.817 | 18.701 | 21.020 | 22.642 | 22.640 | 23.726 | 23.269 | 26.632 | 24.811 |
| **HPGD** | U | U | 25.089 | 21.813 | 26.779 | 26.765 | 26.272 | 27.321 | 21.760 | U | U | 35.454 | U | U | U | U |
| **HRH1** | 27.811 | U | 24.135 | 22.404 | 23.704 | 22.263 | 22.976 | 29.007 | 22.832 | U | 24.147 | 26.385 | 27.336 | U | U | 28.676 |
| **HRH2** | 27.985 | U | 24.289 | 22.343 | 24.353 | 23.291 | 24.027 | 26.450 | 22.003 | 28.745 | 24.087 | 25.622 | U | 27.346 | 28.204 | 28.260 |
| **HRH3** | U | U | U | U | U | U | U | U | U | U | U | U | U | U | U | U |
| **HTR3A** | U | U | 28.303 | 27.436 | U | U | U | U | 39.101 | U | U | U | U | U | U | U |
| **HTR3B** | U | U | U | U | U | U | U | U | U | U | U | 29.205 | U | U | U | U |
| **ICAM1** | 29.520 | 30.364 | 27.004 | 24.797 | U | 27.427 | 30.059 | 29.138 | 22.133 | 26.761 | U | 27.424 | U | 28.151 | U | U |
| **IL13** | U | U | U | U | U | U | U | U | 27.521 | U | U | 33.930 | U | U | U | U |
| **IL1R1** | U | 30.038 | U | 27.869 | U | 39.801 | U | U | 27.492 | 28.665 | U | 39.608 | U | U | U | U |
| **IL1R2** | 30.965 | 40 | 26.194 | 23.970 | U | 27.080 | 31.292 | U | 22.121 | 26.193 | 28.136 | 26.538 | 28.720 | 27.145 | 29.972 | 29.343 |
| **IL1RAPL2** | U | U | U | U | U | U | U | U | U | U | U | U | U | U | U | U |
| **IL1RL1** | U | U | U | 27.397 | U | 36.476 | U | U | U | U | U | 32.551 | U | U | U | U |
| **IL2RA** | U | U | U | U | U | U | U | U | U | U | U | U | U | U | U | U |
| **IL2RB** | U | U | U | U | U | U | U | U | 28.906 | 31.367 | U | U | U | U | U | U |
| **IL2RG** | 28.668 | 28.619 | 26.819 | 23.981 | U | 26.526 | 28.770 | 28.945 | 22.715 | 25.839 | 28.776 | 27.054 | U | 28.697 | 28.941 | U |
| **ITGAL** | U | 27.834 | U | 25.714 | U | 27.996 | U | U | 25.364 | 24.795 | U | U | U | U | U | U |
| **ITGAM** | 28.239 | U | 28.607 | 25.549 | U | 26.660 | U | 30.392 | 24.336 | 27.248 | U | U | 29.552 | U | U | U |
| **ITGB1** | 28.781 | U | 27.614 | 25.289 | U | 29.296 | U | U | 23.650 | U | 28.967 | 27.729 | U | U | U | U |
| **ITGB2** | 26.240 | 25.994 | 23.216 | 21.342 | U | 23.894 | 29.732 | 24.544 | 20.161 | 22.775 | 27.856 | 24.109 | U | 26.877 | U | 26.638 |
| **KLK14** | U | U | U | 27.195 | U | U | U | U | 27.532 | U | U | U | U | U | U | U |
| **KLK15** | U | U | U | 28.817 | U | U | U | U | 33.542 | U | U | U | U | 20.602 | U | U |
| **KLK1** | 28.525 | U | 23.590 | 18.569 | U | 27.754 | 26.061 | 25.679 | 19.749 | 20.979 | 26.168 | 24.937 | 27.625 | 23.521 | U | 29.324 |
| **KLK2** | U | U | U | 29.747 | U | U | U | U | 26.311 | U | U | U | U | 29.502 | U | U |
| **KLK3** | U | 35.912 | U | 36.293 | U | U | U | U | 30.475 | 34.516 | U | 35.616 | U | U | U | U |
| **KLKB1** | U | U | U | U | U | U | U | U | 30.429 | 25.480 | U | 32.463 | U | U | U | U |
| **KNG1** | U | U | U | U | U | U | U | U | 30.940 | U | U | 36.101 | U | U | U | U |
| **LTA4H** | 25.999 | 24.679 | 24.619 | 20.930 | U | 25.045 | 27.270 | 26.358 | 21.460 | 23.809 | 27.817 | 24.932 | 27.889 | 28.297 | U | U |
| **LTB4R2** | U | U | 26.005 | 24.705 | 24.498 | 24.790 | 25.212 | 29.712 | 24.888 | 33.335 | 27.270 | U | 28.839 | U | U | U |
| **LTB4R** | 28.511 | 29.042 | 24.273 | 23.222 | 24.304 | 23.317 | 24.122 | 27.324 | 23.359 | 34.116 | 25.758 | 27.251 | 29.024 | 29.481 | U | 27.716 |
| **LTC4S** | U | U | 28.943 | 25.077 | U | U | 28.491 | U | 26.052 | 28.429 | U | 34.441 | U | U | U | U |
| **MAPK14** | U | 28.243 | 23.022 | 21.361 | U | 26.100 | 27.141 | 26.743 | 21.519 | 23.977 | 24.903 | 25.276 | U | 27.973 | U | U |
| **MAPK1** | 26.270 | 27.668 | 24.399 | 22.259 | 33.006 | 26.570 | 28.726 | 31.864 | 21.993 | 24.815 | U | 26.701 | 29.224 | 27.214 | 29.133 | U |
| **MAPK3** | 26.611 | 24.901 | 24.075 | 21.795 | 27.296 | 24.780 | 27.150 | 27.549 | 21.947 | 24.971 | 29.329 | 26.851 | 29.010 | 28.572 | 29.617 | U |
| **MAPK8** | 27.509 | 26.792 | 23.940 | 22.149 | U | 25.550 | U | 27.140 | 21.733 | 24.015 | 27.458 | 26.019 | U | 25.615 | U | U |
| **MC2R** | U | U | 23.961 | 22.703 | 23.831 | 23.813 | 24.376 | 26.602 | 22.886 | 25.831 | 25.433 | 33.749 | 29.156 | 27.602 | U | 28.297 |
| **NFKB1** | 27.870 | U | 23.944 | 24.124 | U | 26.910 | U | 28.081 | 23.218 | 25.936 | U | 27.704 | U | U | U | U |
| **NOS2A** | U | U | 26.251 | 24.811 | U | U | U | U | 25.971 | 27.135 | U | 35.074 | U | U | U | U |
| **NR3C1** | U | U | 25.978 | 24.061 | U | 27.626 | U | U | 23.122 | 25.913 | U | 26.818 | U | 28.491 | U | U |
| **PDE4A** | 31.584 | 27.653 | 26.765 | 25.492 | U | U | U | U | 24.900 | 26.547 | U | 27.362 | 27.930 | U | U | U |
| **PDE4B** | 26.236 | 28.537 | 24.925 | 23.549 | 28.898 | 25.488 | 27.646 | U | 22.287 | 25.760 | 26.888 | 26.010 | 29.304 | 28.200 | 30.450 | 28.454 |
| **PDE4C** | U | U | U | U | U | U | U | U | U | U | U | U | U | U | U | U |
| **PDE4D** | 30.137 | 31.092 | 29.430 | U | U | 29.970 | U | U | 26.572 | 28.714 | U | U | 30.339 | 30.266 | U | U |
| **PLA2G10** | U | U | U | U | U | U | U | U | 27.109 | 26.223 | U | U | U | U | U | U |
| **PLA2G1B** | U | U | U | U | U | U | U | U | U | 28.979 | U | U | U | U | U | U |
| **PLA2G2A** | 25.981 | 26.328 | 23.682 | 22.775 | U | 30.504 | 29.662 | 28.310 | 26.572 | U | 27.868 | 26.540 | U | 31.069 | U | U |
| **PLA2G2D** | U | U | U | U | U | U | U | U | U | U | U | U | U | U | U | U |
| **PLA2G4C** | U | U | U | U | U | U | U | U | U | U | U | 36.050 | U | U | U | U |
| **PLA2G5** | U | U | U | U | U | U | U | U | U | U | U | U | U | U | U | U |
| **PLA2G7** | U | U | U | U | U | U | U | U | U | U | U | U | U | U | U | U |
| **PLCB2** | 28.710 | 29.309 | 30.671 | 27.234 | U | 27.826 | U | U | 25.490 | 30.223 | U | 30.889 | U | U | U | U |
| **PLCB3** | 28.604 | 28.150 | U | 25.769 | U | U | U | U | 26.941 | 27.871 | U | U | U | U | U | U |
| **PLCB4** | 27.791 | 27.665 | 25.641 | 24.505 | U | U | U | U | 25.831 | 28.698 | 30.503 | U | U | 27.974 | U | U |
| **PLCD1** | U | U | 29.011 | 27.010 | U | U | U | U | 25.887 | U | U | 28.984 | U | 28.706 | U | U |
| **PLCE1** | U | U | U | U | U | U | U | U | U | U | U | U | U | U | U | U |
| **PLCG1** | U | U | U | 24.925 | U | U | U | U | 24.770 | 27.331 | U | U | U | U | U | U |
| **PLCG2** | 29.991 | U | 28.007 | 26.974 | U | 27.541 | U | U | 25.257 | 29.509 | 30.004 | 29.130 | U | U | U | U |
| **PTAFR** | U | U | 24.732 | 22.723 | 24.135 | 23.617 | 23.317 | 27.133 | 23.058 | 26.993 | 25.967 | 27.315 | 28.314 | 28.385 | 28.351 | 24.989 |
| **PTGDR** | U | U | U | U | U | U | U | U | 28.445 | 34.240 | 7.146 | 38.116 | U | U | U | 39.250 |
| **PTGER2** | U | U | U | 27.729 | U | U | U | U | U | U | U | 31.176 | U | U | U | U |
| **PTGER3** | U | U | U | 29.139 | U | U | U | U | 27.041 | U | U | 29.168 | U | U | U | U |
| **PTGFR** | U | 26.878 | U | U | U | U | U | U | 34.234 | U | U | U | U | U | U | U |
| **PTGIR** | U | U | U | U | U | U | U | U | U | U | U | U | U | U | U | 35.205 |
| **PTGIS** | U | U | U | U | U | U | U | U | U | U | U | U | 19.392 | 22.052 | U | U |
| **PTGS1** | U | U | U | 28.811 | U | U | U | U | 29.776 | U | U | U | U | U | U | U |
| **PTGS2** | 24.209 | 24.888 | 22.274 | 20.476 | 29.388 | 23.524 | 27.101 | 24.681 | 18.641 | 22.033 | 26.140 | 24.189 | 25.959 | 25.943 | 27.232 | 24.626 |
| **TBXA2R** | U | U | U | U | U | U | U | U | 30.361 | U | U | U | U | U | U | U |
| **TBXAS1** | 29.982 | U | 26.376 | 25.036 | U | 26.395 | U | U | 23.353 | 27.882 | U | 27.113 | U | 30.132 | U | U |
| **TNF** | U | U | 24.939 | 24.010 | U | 27.685 | 22.255 | 27.688 | 24.051 | 26.951 | U | 28.154 | U | U | U | U |
| **TNFRSF1A** | 25.800 | 25.862 | 23.054 | 21.642 | 28.214 | 23.121 | 25.461 | 26.042 | 19.868 | 24.086 | 26.817 | 23.127 | U | 26.462 | 28.961 | 27.621 |
| **TNFRSF1B** | 28.210 | 28.487 | 25.363 | 23.510 | U | 25.056 | 28.289 | 26.380 | 20.873 | 24.756 | 26.410 | 26.261 | U | 27.335 | U | U |
| **TNFSF13B** | 30.447 | 40 | 27.164 | 24.835 | U | 25.380 | 30.192 | 29.520 | 23.105 | 25.760 | 30.012 | 28.198 | U | 28.043 | 27.937 | U |
| **VCAM1** | U | U | U | U | U | U | U | U | 30.645 | U | U | U | U | U | U | U |

**Supplemental Table S3.** Cycle threshold values from real time PCR for 7 non-fighter controls for 96 genes from the Taqman Human Inflammation array. U= undetermined

|  | **con1** | **con2** | **con3** | **con4** | **con5** | **con6** | **con7** |
| --- | --- | --- | --- | --- | --- | --- | --- |
| **18S** | 25.155 | 23.869 | 21.733 | 22.992 | 24.568 | 22.556 | 26.524 |
| **A2M** | 29.267 | U | U | U | U | U | U |
| **ACTB** | 19.884 | 21.997 | 18.743 | 18.945 | 24.449 | 21.259 | 21.867 |
| **ADRB1** | 25.590 | 29.595 | 28.760 | 32.222 | 29.578 | 25.862 | U |
| **ADRB2** | 23.322 | 26.867 | 25.808 | 26.933 | 27.941 | 26.173 | 26.553 |
| **ALOX12** | 24.976 | 27.564 | 24.652 | 25.787 | 25.275 | 24.113 | 22.308 |
| **ALOX5** | 23.278 | 26.208 | 22.732 | 22.792 | 27.084 | 25.875 | U |
| **ANXA1** | 18.950 | 23.575 | 18.578 | 21.090 | 21.421 | 18.702 | 18.342 |
| **ANXA3** | 23.252 | 27.733 | 23.694 | 26.623 | U | 25.590 | 25.664 |
| **ANXA5** | 23.575 | 24.750 | 22.591 | 25.130 | 27.556 | 24.727 | 22.759 |
| **B2M** | 18.365 | 22.195 | 18.052 | 18.750 | 25.973 | 21.251 | 18.362 |
| **BDKRB1** | 24.468 | 27.570 | 26.064 | 30.861 | 27.983 | 26.736 | U |
| **BDKRB2** | U | U | 25.860 | U | 25.360 | U | U |
| **CACNA1C** | U | U | U | U | U | U | U |
| **CACNA1D** | 28.294 | U | U | U | U | 29.726 | U |
| **CACNA2D1** | U | U | U | U | U | U | U |
| **CACNB2** | U | U | U | 29.429 | U | U | U |
| **CACNB4** | 27.810 | 29.894 | 26.189 | 29.403 | U | 28.124 | 26.826 |
| **CASP1** | 25.113 | 27.673 | 24.775 | 26.591 | U | 27.496 | 23.874 |
| **CD40** | U | 28.041 | 28.278 | U | U | U | U |
| **CD40LG** | U | U | U | U | U | U | U |
| **CES1** | 28.807 | U | U | U | U | U | U |
| **CYSLTR1** | 24.120 | U | 26.287 | 28.523 | U | 26.247 | 27.437 |
| **GAPDH** | 20.316 | 22.743 | 19.503 | 22.029 | 25.407 | 22.134 | 22.421 |
| **HPGD** | 24.222 | 35.738 | 24.869 | U | 25.702 | 25.281 | 24.069 |
| **HRH1** | 23.600 | 28.548 | 26.795 | U | 29.890 | 26.793 | U |
| **HRH2** | 23.495 | 33.452 | 25.240 | 26.411 | 27.041 | 27.416 | U |
| **HRH3** | U | U | U | U | U | U | U |
| **HTR3A** | U | U | U | U | U | U | U |
| **HTR3B** | U | U | U | U | U | U | U |
| **ICAM1** | 29.189 | 28.469 | 25.260 | 24.593 | 30.562 | 26.688 | 28.827 |
| **IL13** | U | U | U | U | U | U | U |
| **IL1R1** | 27.097 | U | U | U | U | 27.032 | U |
| **IL1R2** | 25.565 | 27.741 | 25.479 | 25.494 | 28.812 | 28.980 | 27.415 |
| **IL1RAPL2** | U | U | U | U | U | U | U |
| **IL1RL1** | U | U | U | 24.637 | U | U | 30.513 |
| **IL2RA** | U | U | U | U | U | U | U |
| **IL2RB** | U | U | U | U | U | U | U |
| **IL2RG** | 25.620 | 27.692 | 26.405 | 24.162 | U | U | U |
| **ITGAL** | 27.550 | 27.749 | U | 27.983 | U | U | U |
| **ITGAM** | 28.905 | 27.180 | 26.093 | 27.091 | U | 28.950 | U |
| **ITGB1** | 25.856 | U | 25.868 | 27.984 | U | U | 25.020 |
| **ITGB2** | 23.768 | 25.114 | 22.522 | 22.431 | 26.690 | 25.269 | U |
| **KLK14** | 29.275 | 29.491 | 27.349 | U | U | 27.759 | U |
| **KLK15** | U | U | U | U | U | 26.836 | U |
| **KLK1** | 23.124 | 26.321 | 23.103 | 27.121 | 27.590 | 24.460 | U |
| **KLK2-** | U | U | U | U | U | U | U |
| **KLK3-** | U | U | U | U | U | U | 21.794 |
| **KLKB1** | U | U | U | U | U | U | U |
| **KNG1** | U | U | U | U | U | U | U |
| **LTA4H** | 22.074 | 27.437 | 23.451 | 26.522 | U | 26.944 | 25.825 |
| **LTB4R2** | 26.494 | 28.825 | 29.146 | 28.084 | U | 26.773 | U |
| **LTB4R** | 25.364 | 27.501 | 26.258 | 27.913 | 27.585 | 26.485 | U |
| **LTC4S** | 28.644 | U | U | U | U | U | U |
| **MAPK14** | 24.837 | 26.464 | 24.557 | 25.724 | U | 26.172 | U |
| **MAPK1** | 24.895 | 27.036 | 25.118 | 25.482 | 28.858 | 26.451 | 27.568 |
| **MAPK3** | 24.312 | 26.864 | 24.435 | 24.498 | 26.784 | 25.101 | 25.165 |
| **MAPK8** | 23.425 | 26.260 | 23.708 | 24.835 | U | 24.816 | 23.775 |
| **MC2R** | 23.297 | 27.941 | 26.667 | U | 28.280 | 26.615 | U |
| **NFKB1** | 24.348 | 27.824 | 25.421 | 26.666 | U | 27.197 | 25.498 |
| **NOS2A** | U | U | U | U | U | U | U |
| **NR3C1** | 25.774 | U | 25.544 | 26.340 | U | U | 28.130 |
| **PDE4A** | U | U | 26.809 | U | U | 28.129 | 23.766 |
| **PDE4B** | 24.125 | 27.298 | 23.972 | 24.942 | 27.882 | 27.126 | 24.168 |
| **PDE4C** | U | U | U | U | U | U | U |
| **PDE4D** | 28.302 | U | 27.254 | 30.195 | U | U | U |
| **PLA2G10** | U | U | U | U | U | U | U |
| **PLA2G1B** | U | U | U | U | U | 28.877 | U |
| **PLA2G2A** | 26.318 | 32.676 | 27.494 | 26.761 | 28.216 | 28.244 | 23.902 |
| **PLA2G2D** | U | U | U | U | U | U | U |
| **PLA2G4C** | U | U | U | 31.495 | U | U | U |
| **PLA2G5** | U | U | U | U | U | U | U |
| **PLA2G7** | U | U | U | U | U | U | U |
| **PLCB2** | U | 34.807 | 26.902 | 29.099 | U | U | U |
| **PLCB3** | U | 32.629 | 29.318 | 28.051 | U | U | 25.747 |
| **PLCB4** | 26.167 | U | 26.668 | 27.747 | U | 27.462 | 25.770 |
| **PLCD1** | 28.423 | U | 26.356 | U | U | U | U |
| **PLCE1** | U | U | 29.774 | U | U | U | U |
| **PLCG1** | 26.801 | 27.439 | 26.515 | U | 27.370 | U | 25.445 |
| **PLCG2** | 27.413 | U | 26.980 | U | U | U | 25.956 |
| **PTAFR** | 24.320 | 28.066 | 24.630 | 25.731 | 26.802 | 25.617 | U |
| **PTGDR** | 33.837 | U | U | U | U | U | U |
| **PTGER2** | U | U | U | 29.233 | U | U | U |
| **PTGER3** | U | U | U | U | U | U | U |
| **PTGFR** | U | U | U | U | U | U | U |
| **PTGIR-** | U | U | U | 38.266 | U | U | U |
| **PTGIS** | U | U | U | U | U | U | U |
| **PTGS1** | U | U | U | U | U | 28.450 | U |
| **PTGS2** | 21.651 | 28.403 | 22.502 | 21.508 | U | 24.935 | 24.518 |
| **TBXA2R** | U | U | U | U | U | U | U |
| **TBXAS1** | 27.151 | U | 25.779 | 25.986 | U | 27.984 | U |
| **TNF** | 24.776 | U | 24.350 | 25.101 | 36.909 | U | U |
| **TNFRSF1A** | 24.723 | 26.017 | 22.287 | 22.525 | 27.983 | 26.185 | 23.495 |
| **TNFRSF1B** | 24.964 | 25.456 | 24.067 | 22.470 | 28.420 | 24.652 | U |
| **TNFSF13B** | 24.570 | 29.460 | 25.040 | 28.112 | U | 28.640 | 23.928 |
| **VCAM1** | U | U | U | U | U | U | U |

**Supplemental Table S4**. Most Common Genetic Pathways and Key Genes Associated with TBI.

**Supplemental Figure S1.** Western blot analysis of the primary antibodies including CD63, CD81, CD9, and heat shock protein 70(hsp70) from System Biosciences EXOABPKIT-1, apoA-I (Santa Cruz Biotechnology), GAPDH (Invitrogen), PDCD6IP (ThemoFisher Scientific).**
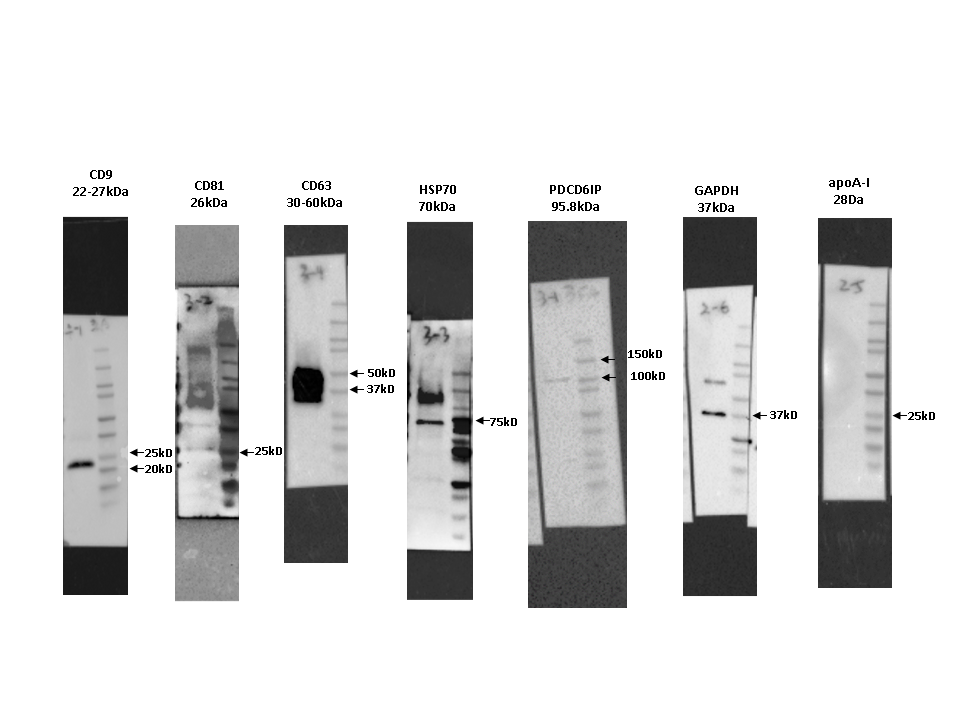
**

**Supplemental Figure S2**. Nanosight analysis of the three participant groups, mean size (nm) and concentration with standard deviation, of fighter EV pre-samples (n=8), post-samples (n=8) and control EV samples (n=7).
